# The 2022 ENCR Recommendations on recording and reporting of urothelial tumours of the urinary tract

**DOI:** 10.3389/fonc.2022.1046239

**Published:** 2022-11-23

**Authors:** Jaume Galceran, David Parada, Michael Eden, Rosario Tumino, Anne Yvonne Warren, Carmen Martos, Luciana Neamtiu, Otto Visser, Laetitia Daubisse-Marliac

**Affiliations:** ^1^ Cancer Epidemiology and Prevention Service, Hospital Universitari Sant Joan de Reus, Reus, Spain; ^2^ Institut d’Investigacions Sanitàries Pere Virgili (IISPV), Reus, Spain; ^3^ Faculty of Medicine and Health Sciences, Universitat Rovira i Virgili, Reus-Tarragona, Spain; ^4^ Pathology Service, Hospital Universitari Sant Joan de Reus, Reus, Spain; ^5^ NHS Digital, Cambridge, United Kingdom; ^6^ Cancer Registry and Histopathology Department, Provincial Health Authority (ASP), Ragusa, Italy; ^7^ Cambridge University Hospitals NHS Foundation Trust, Cambridge, United Kingdom; ^8^ European Commission, Joint Research Centre (JRC), Ispra, Italy; ^9^ Netherlands Comprehensive Cancer Organization (IKNL), Utrecht, Netherlands; ^10^ Claudius Regaud Institute, IUCT-O, Tarn Cancer Registry, Toulouse, France; ^11^ CERPOP, Toulouse University, Inserm UMR 1295, UPS, Toulouse, France; ^12^ FRANCIM, Network of French Cancer Registries, Toulouse, France; ^13^ University Hospital Center, IUCT-O, Cancer Coordination Center, Toulouse, France

**Keywords:** urothelial tumors, recommendations, bladder cancer, recording, reporting, registration practices, cancer registry, Europe

## Abstract

An updated European Network of Cancer registries (ENCR) Recommendations on Recording and Reporting of Urothelial Tumours of the Urinary Tract had been published in 2022. After the publication by the ENCR of the “Recommendations for coding bladder cancers” in 1995, knowledge about the biology and pathology of urinary tract tumors and their classification has varied and increased substantially. On the other hand, several studies have shown that cancer registries use different definitions, criteria for inclusion and coding of urothelial tumors. This great variability among registries affects not only the criteria for recording (registration, coding and classification) but also the criteria of reporting (counting in the statistics of incidence and survival) urinary tract tumors. This causes difficulties in the data comparability from different registries. Recording and reporting of urothelial tumors requires the application of standard criteria that must take into account the combination of the multiple aspects as the primary topography, the histological type, the grade, the extent of invasion, the multi-centricity, the progressions and the time interval between tumors. This led to the creation of a Working Group of the ENCR that developed these recommendations on the recording and reporting of urothelial tumors of the urinary tract. This article reports these recommendations and the rationale for each.

## Introduction

In 1995 the European Network of Cancer Registries (ENCR) distributed the “Recommendations for coding bladder cancers” (1). These recommendations were elaborated because of the special characteristics of urothelial tumors and, especially, the difficulties of clinicians and pathologists to correctly determine their morphology, level of invasion and grade and, which makes it impossible to correctly and precisely classify them.

Among the characteristics that make it difficult to record, code and report urothelial tumors are their multicentricity, their great capacity for recurrence and progression, difficulties in correctly determining their grade and level of invasion, and the existence of variants and types that can be confused with other tumors.

After the publication of these Recommendations, knowledge of the biology and pathology of urinary tract tumors has increased substantially and, therefore, their classification has been modified (2, 3). On the other hand, several studies have shown that cancer registries use different definitions, criteria for inclusion and coding of urothelial tumors (4). A recently published study confirms that this variability is still relevant today (5). This wide variability among registries affects not only the criteria for recording (registration, coding and classification) but also the criteria of reporting (counting in the statistics of incidence and survival) urinary tract tumors. This makes it difficult to compare urothelial tumor burden between cancer registries.

The recording and reporting of urothelial tumors requires the application of standard criteria. The combination of multiple aspects must be taken into account: the primary topography, the histological type, the grade, the extent of invasion, the multi-centricity, the recurrences and progressions and the time interval between tumors, the difficulties in the obtaining of the result of biopsies, the recording stage, the existence of tumors diagnosed before the registry’s period of recording, the residence of patients at the time of diagnosis of each tumor and the standard criteria for multiplicity. All this led to the creation of a new ENCR Working Group that has reviewed and updated the ENCR Recommendations published in 1995. These new recommendations were published/distributed in June 2022 under the title “ENCR Recommendations on Recording and Reporting of Urothelial Tumours of the Urinary Tract” and European population-based cancer registries must apply them to all urothelial tumors with an incidence date of 1st January 2022 or later (6).

These recommendations are based on current knowledge about the biology, anatomical pathology and epidemiology of urinary tract tumors reflected in the fourth edition of the WHO Classification of Tumors of the Urinary System and Male Genital Organs of 2016 (2) and also in new knowledge on urothelial tumors published more recently (7–9). Although WHO 2016 classification has been used, these recommendations include all the aspects listed in the previous paragraph for recording and reporting these tumors in a harmonized way in the European cancer registries.

These recommendations will enable population-based cancer registries to improve the quality of their data and the comparability of incidence and survival data, while providing useful information to clinicians and policymakers. This document reports these recommendations and the rationale for each one of them.

## Methods

In 2017, the cancer registries of Tarn (France) and Tarragona and Girona (Spain) launched a survey to European cancer registries on the practices of registration, coding and reporting of urothelial tumors. For example, in cases in which the tumor presented various levels of progression. The survey was answered by 42 registries. The conclusions of the survey were that there was an urgent need to define clear rules for the registration these tumors. As an example, in cases where the tumor had various levels of progression from a low-grade non-invasive tumor to an invasive tumor, 8 recorded only the first tumor, one recorded only the last (invasive), 13 recorded the first (low-grade non-invasive) and the last; 11 recorded combinations that included the first and the last, and 9 recorded all tumors. In relation to reporting, there was also great variability: 18 reported only the first, 13 reported only the last, 10 reported combinations of one or several tumors, and one did not report any tumor. In addition to questions on inclusion (recording) and reporting criteria, the survey also included questions on coding composite tumors such as urothelial carcinomas with squamous, adenocarcinomatous or neuroendocrine component, and neuroendocrine carcinoma with urothelial carcinoma. In this aspect, the degree of discordance was lower.

In June 2018, the ENCR launched an offer of expressions of interest from member registries to join an Urothelial Cancers Task Force that would include both cancer registry and clinical representation. The aims of the Working Group (WG) were to address the difficulties in the registration of urothelial cancers and to update the ENCR recommendations published in 1995. The new ENCR Recommendations would improve incidence and survival data comparability across different European registries and countries.

Once the WG was established, a first meeting was held by teleconference on December 5, 2018. On July 12, 2019, a first face-to-face meeting of the Working Group was held at the Join Research Center (JRC) in Ispra, Italy, at which it was decided to update and to draft the ENCR Recommendations. A second face-to-face meeting was also held in Ispra on November 8, 2019. After the second meeting, the WG continued its work virtually, introducing modifications to previous versions of the document.

During the meetings and during the virtual work of the Working Group, each of the decisions on recording and coding issues were made by consensus of at least 7 of the 8 members of the group.

**Figure 1:**
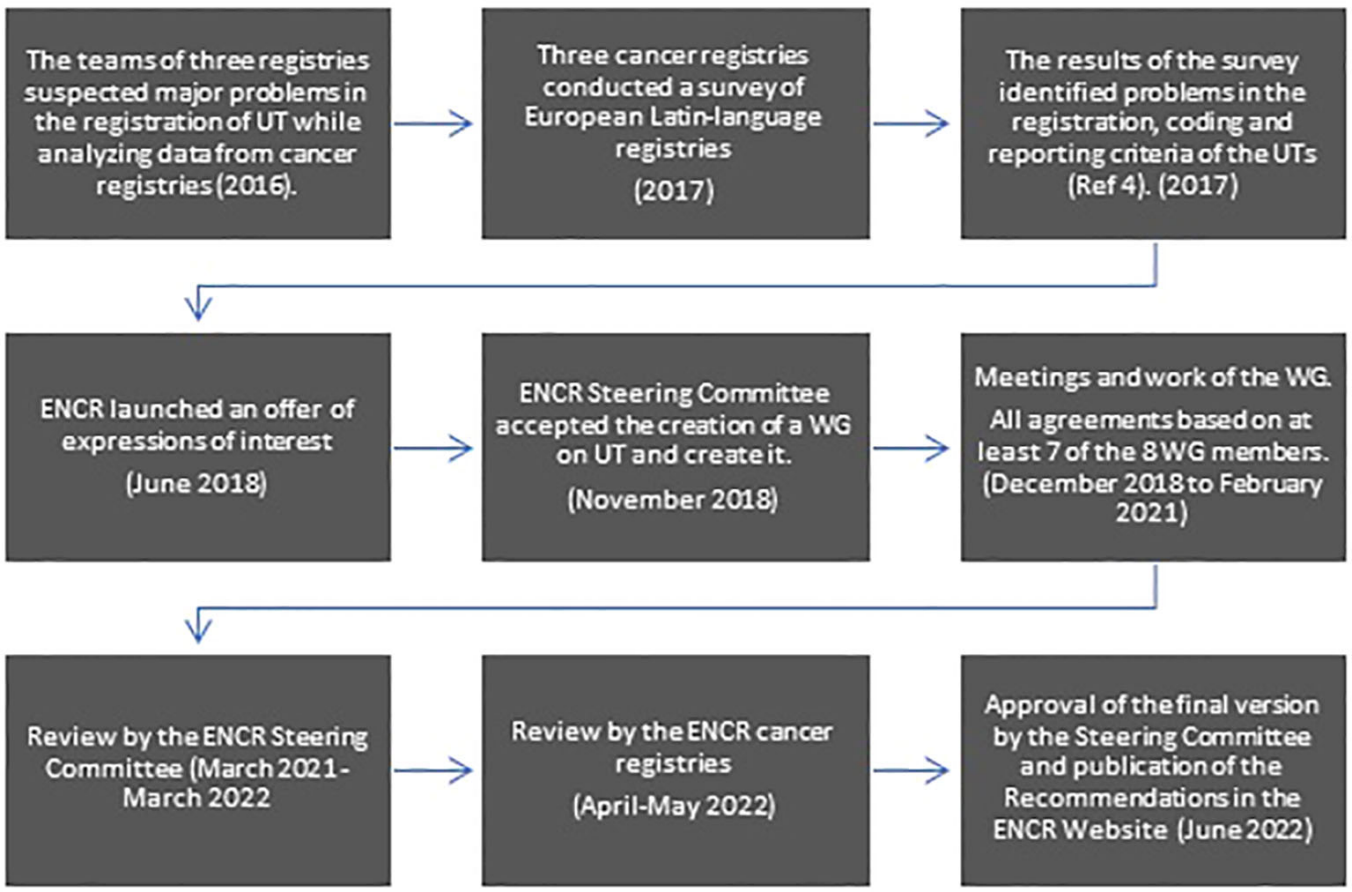
Scheme of the process of preparing the ENCR Recommendations on Urothelial Cancers.

Once the WG finalized the draft Recommendations, it was sent to the ENCR Steering Committee, which reviewed it and proposed some modifications. Once the Working Group and the Steering Committee agreed on the document, it was sent to all ENCR members for revision and feedback. Some registries sent their comments and asked for clarifications. All the questions asked were answered and some of the registries’ proposed modifications were introduced. Finally, on June 8, 2022, the Steering Committee approved the final version of the Recommendations that were published on the ENCR website a few days later (https://encr.eu/sites/default/files/Recommendations/ENCR%20Recommendation_UT_Jun2022_EN.pdf). **Figure 1** shows the scheme of this process from the offer of expression of interest to the publication of the Recommendations.

## Results and discussion

In cancer registration and especially in the registration of some types of tumors with high rates of recurrence and progression such as urothelial tumors of the urinary tract, it is important to differentiate between **recording** (registration) and **reporting** (counting) tumors. A cancer registry can record several tumors of the urothelium (of different site, grade or invasion) of the same patient but according to international criteria and for the purposes of comparability, only one or a part of them is actually reported.

## Recommendations for recording urothelial tumors

### The recommendations for recording of urothelial tumors are based on three general principles

First, these Recommendations apply to all urothelial tumors (transitional cell tumors) and their variants regardless of tumor topography (renal pelvis, ureter, urinary bladder, or urethra –International Classification of Diseases for Oncology, Third Edition (ICD-O-3) codes C65 to C68–). Therefore, they apply to the pure urothelial carcinomas, to urothelial carcinomas with divergent (squamous, glandular, trophoblastic and other) differentiation, and to all other variants (nested, microcystic, lymphoepithelioma-like, plasmacytoid/signet ring cell, sarcomatoid, giant cell, lipid-rich, clear cell and poorly differentiated) of urothelial carcinomas. Sarcomas and other histologic types of cancer (e.g., adenocarcinomas, squamous cell carcinomas, or neuroendocrine tumors) of the urinary tract are not included in these recommendations, although they do occur in the urinary tract and should also be recorded by registries.

Secondly, in order to correctly record and code urothelial tumors, it is essential to have access to pathological examinations (reports) since knowledge of the topography, morphological type, behavior and grade of the tumor is required. The non-existence or non-availability of anatomopathological reports prevents, in many cases, knowing whether the tumor should be registered and, in all cases, accurate coding of some variables (morphology, behavior, grade…).

Third, although in cancer registration, the usual definition of synchronous tumors includes all tumors of the same location that appear in a period of less than or equal to 3 months, these specific recommendations for urothelial tumors define synchronous tumors of the same location and laterality as those that present in a period of less than or equal to 4 months. This criterion also applies to urothelial tumors whose resection is performed in two phases since, in many of these cases, the initial resections are not complete or the second revision is sometimes delayed, particularly in elderly patients.

## Criteria for the inclusion (registration) of urothelial tumors

In the following paragraphs, the 11 rules or criteria for the inclusion (registration) of urothelial tumors in the cancer registry are described. Each of the inclusion criteria is indicated in italics and indented as they are in the European Network of Cancer Registries Recommendations document (6).

### Types of tumors to be included

Cancer registries must record all invasive and non-invasive urothelial carcinomas including those without histological confirmation. Obviously, the urothelial concept includes any type of urothelial carcinoma and any of its variants.

The 4^th^ and 5^th^ editions of the WHO Classification of Tumours of the Urinary System (2, 3) allow a clear differentiation between malignant tumors (invasive or not) and non-malignant tumors. According this new WHO Classification, papillary urothelial neoplasms of low malignant potential (PUNLMP), urothelial papillomas, inverted urothelial papillomas, urothelial proliferation of uncertain malignant potential and urothelial dysplasia are not considered malignant, and are therefore not recommended for registration in a cancer registry. However, cancer registries that for whatever reason are interested in any of these entities may register them if they wish, but they should never be included in the *incidence computation.*

“*The following types of tumors arising in the urinary tract must be recorded:*

*1. Non-invasive papillary urothelial carcinoma, low-grade*

*2. Non-invasive papillary urothelial carcinoma, high-grade*

*3. Urothelial carcinoma *in situ* (carcinoma *in situ*)*

*4. All invasive carcinomas*

*5. Tumour with histologic examination but invasion cannot be assessed*

*6. Tumour with cytological examination only (see rule 2.b, page 6)*

*7. Tumour with no microscopic confirmation (see rule 2.c, page 6)”*

### Multiples sites

The International Rules for Multiple Primary Cancer edited jointly by the International Agency for Research on Cancer, the World Health Organization, the International Association of Cancer Registries and the European Network of Cancer Registries in 2004 (10) are for “reporting” data on cancer incidence and survival, so that cancer risk and outcome are comparable between different populations. The same Rules indicate that for collection, it is recommended that registries collect and register more detailed data and, in fact, cancer registries use different rules for defining multiple primaries when registering cancer cases. Such cases should be collapsed to conform to the international rules for analysis.

The WG that prepared these updated ENCR Recommendations considered that, in order to be able to analyze many aspects of these tumors, it is necessary to have information on all tumors with different three-digit ICD-O-3 topography. Therefore, the recommendation is

*“if a patient presents with several (synchronous or metachronous) urothelial tumors in different sites, record all tumours of different three-digit sites (C65-C68) and laterality (if renal pelvis or ureter). If a metachronous tumor is diagnosed in the ureter or urethra after cystectomy, it should not be recorded if it has arisen at the surgical margin because it should be considered as a local recurrence of the removed tumor in the urinary bladder except if it is a progression.”*

### Progressions

A characteristic of urothelial carcinomas is their high capacity for recurrence and progression. Reported 5-year rates of non-muscle-invasive bladder carcinoma recurrence range from 50% to 70% and reported 5-year rates of progression range from 10% to 30%. Factors associated with recurrence and progression include, among others, high grade, high stage, large tumor size, multifocality, high number of previous recurrences and presence of concomitant carcinoma *in situ* (11), and histological variants. Tumor grade, stage, and carcinoma *in situ* are the most important variables for progression (12) Taking into account this ability of urothelial tumors to progress, and their different prognosis depending on their grade, level of invasion and morphology, it has been considered necessary that cancer registries record progressions in order to be able to correctly compare survival among different populations.

Studies have suggested that invasive urothelial tumors develop along at least two molecular pathways, *via* either high-grade papillary tumors or carcinoma *in situ* (7, 13). For this reason, when a new urothelial tumor is diagnosed in a patient who already has previous tumors, it can be difficult to define whether or not the new tumor represents progression. Therefore, in these recommendations the process of progression was determined not on the basis of the molecular pathway but on the basis of the severity of the tumor and its ability to progress further. Thus, carcinoma *in situ* was considered as progression of high-grade non-invasive carcinoma and the recommendation was defined as follows:

*“If a patient presents with several urothelial tumors in the same three-digit topographical site that includes some progression of the disease, register the first tumor and then subsequently only those tumours that represent a chronological progression. The following series shows the order that represent a progression:*

*Non-invasive, low grade (TaG1) → Non-invasive, high grade (TaG3) → *In situ* (Tis) → Invasive, superficial (T1) → Muscle-invasive (T2+).*

*Due to the special characteristics of urothelial tumours, the recording of the different stages should be done for these tumours in order to know their progression. Remember that all known steps of this progression should be recorded. Therefore, for example, the recording of a T2+ invasive tumor does not replace the recording of a T1 invasive tumor if the latter is known.”*

### Recurrences

It has already been mentioned that urothelial tumors have a great tendency to present with recurrences and progressions. Multiplicity, tumor size, and prior recurrence rate are the most important variables for recurrence (12). Recurrences do not significantly change the patient’s prognosis. Therefore the fourth rule specifies that:

*“Tumours that represent recurrences (not progressions) with the same or lower level of invasion and degree do not have to be recorded”.*

### Synchronous urothelial tumors of the same site and laterality

Tumor multifocality, that is, the existence of two or more non-contiguous tumor formations separated by a macroscopically non-tumorous tissue area, is common in urothelial carcinomas. In carcinoma *in situ*, involvement of the surface urothelium is usually multifocal (14). In non-invasive urothelial tumors, multifocality is one of the factors determining clinical risk of recurrence and disease progression (2). Due to this characteristic of urothelial tumors, the WG agreed that the presence of multifocality at the same topography has to be registered as a single tumor and if the different tumors have a different level of aggressiveness (grade, level of invasion -T-), the one to be registered is the most aggressive one, to ensure a correct survival analysis. This standard should apply to synchronous tumors and, as discussed in the general principles of urothelial tumor registration, this means all tumors within a maximum period of 4 months between them.

*“If a patient presents with more than one urothelial tumour in the same three-digit topographical site and laterality (if renal pelvis or ureter) in a short period of time (≤4 months – i.e. synchronous–), record only the most aggressive of them (based on the progression scheme in point 3 above) but with the date of diagnosis taken from the first tumour.*

*This criterion also applies to tumours whose resection is performed in two phases. In these cases, the temporal course of clinical investigation should also be considered because sometimes initial resections are not complete or the second look is sometimes delayed, particularly in old patients.”*

### Codes of site in synchronous tumors of bladder:

Two or more tumors may arise synchronously in the bladder, with similar or different aggressiveness. In this case, if the two (or more) tumors are in the same subsite of the bladder, this subsite should be coded, but if the tumors are in different subsites, code C67.8 should be recorded to follow ICD-O criteria on “Tumors involving more than one topographic category or subcategory”.

*“Record synchronous tumors of the bladder using the synchronous tumor rule (rule 5). If the highest level of progression is present in more than one tumour and in more than one subsite (four-digit topography), code the site as C67.8 even if the tumours are not contiguous. If they appear in the same subsite, codify the corresponding subsite.”*

### Synchronous urothelial tumors of different site

Although the International Rules for Multiple Primary Cancers (ICD-O Third Edition) for reporting tumors consider tumors of the renal pelvis (C65), ureter (C66), urinary bladder (C67) and other and unspecified urinary organs (C68.9) as belonging to a single topographic site, it is highly recommended that tumors from different three-digit ICD-O-3 sites be recorded as different tumors. First, the separate registration of the multiple tumors allows registries to better describe the incidence. Second, for survival studies, the knowledge of the existence of multiple tumors and their site is fundamental since the prognosis depends, among other factors, on the primary site where the tumor has developed.

*“If a patient presents with more than one urothelial tumor in different three-digit topographical sites in a short period of time (≤4 months –synchronous–), record each tumor separately, each one with its corresponding topography, morphology, behavior codes and incidence date (do not use grouping code C68.9 for registration purpose)”.*

### Bilateral tumors

Unlike synchronous urothelial tumors of different site, bilateral tumors share the same site code. However, having a single tumor at a paired site (pelvis or ureter) does not carry the same prognosis as having a tumor at each of the paired sites. Furthermore, their aggressiveness (grade, level of invasion, morphological type) may be different. For these reasons, and although only one tumor should be counted for the calculation of incidence, it is recommended that bilateral tumors of the same site be recorded according to the following criteria:

*“If a patient presents with several (synchronous or metachronous) urothelial tumours in both sides of the same paired organ (e.g. right and left pelvis or right and left ureter), record all the tumors of each side of each three digit site following rules 3 to 6 (e.g. 1st urothelial carcinoma in right ureter and its progressions, and 1st urothelial carcinoma in left ureter and its progressions).”*

### Mixed situations of multiplicity, progressions and synchronicity/metachronicity

Due to the multifocality and progressive characteristics of urothelial tumors, there are many possible combinations of multiplicity, progression and temporality. Consider the example of a patient who presents with a mixed combination of multiple synchronous and metachronous urothelial tumors in the same and different three-digit topographies. This patient presented in this chronological order with a “Non-invasive low-grade carcinoma” of bladder (1) followed by a synchronous “Invasive carcinoma” of bladder (2) followed by an “*In situ* carcinoma” of right renal pelvis (3) followed by a “Non-Invasive high-grade carcinoma” of right renal pelvis (4) followed by an “Invasive carcinoma” of bladder (5).

Tumor 1 and tumor 2 are synchronous at the same site, so only the more aggressive, in this case the invasive one should be recorded (with the date of diagnosis of the first tumor). Tumor 3 should be recorded because it appeared in a different site. Tumor 4, on the other hand, should not be recorded because it must be considered a recurrence of tumor 3. Finally, tumor 5 should not be recorded either because it is a recurrence of tumor 2.

*“If a patient presents with a combination of synchronous and metachronous multiple urothelial tumors in the same and/or different three-digit sites, record them according to rules 2 to 8.”*

### First tumor occurring outside the area of registration

In cancer registries it is possible to find cancers of the same topography separated in time. The higher the incidence of a type of cancer and its survival, the more likely it is to find this phenomenon. Colorectal and breast cancers are a good example of this. But, once again, due to their high capacity for recurrence and progression, urothelial carcinomas are the ones that present this phenomenon most frequently, except for non-melanoma skin cancers.

People can change their residence throughout their lives and each tumor is registered in association with the patient’s residence at the time of diagnosis. This may result in a first tumor being diagnosed when the patient resides outside the registry area and the next one(s) being diagnosed when the patient resides in the registry area. In this situation, if we do not record the first tumor, we will not be aware that the second is not an incident case but rather a prevalent one and, therefore, we will mistakenly count it as incident. This will cause an overestimation of the incidence. So, the recording of a first tumor diagnosed outside the area of registration allows the registry to know if a subsequent tumor is a recurrence or progression (recorded but not reported as incident) thus avoiding over-reporting.

*“A patient can move from one residence to another, so place of residence should be related to the tumours and not to the patient. If information is available showing a patient resident in the coverage area of the registry has been previously diagnosed with a urothelial tumor(s) when resident outside the registration area, record all of them (the ones occurring outside the area of registration and the ones diagnosed being resident in the area of the registry) according to rules 2 to 8 (that enables the tumours to be flagged as ‘Extra-regional’ for reporting purposes).”*

### First tumor occurring before the operation period of the registry

A similar situation occurs when the first tumor is diagnosed before the registry operation period. If we do not record the first tumor diagnosed before the registration period, it is impossible to know that successive cancers from the same site are recurrences or progressions and not cancers that should be reported as incident. So, the recording of tumors diagnosed before the period of operation of the registry allows the registry to know whether subsequent tumors should be recorded as progression or recurrence (recorded but not reported as incident) to prevent over-reporting.

*“If information is available showing a patient resident in the coverage area of the registry has been diagnosed with one or more urothelial tumors before the operation period of the registry, record all their tumors (the ones diagnosed before and the one diagnosed after first date of operation of the registry) according to rules 2 to 8.”*

## Recommendations for classification and coding

In the following paragraphs, the recommendations for the coding and classification of urothelial tumors are described. As has been done with the inclusion criteria section, recommendations are indicated in italics and indented as they are in the ENCR Recommendations document (6).

### Classification used in the cancer registries

Cancer registries usually code topography, morphology, behavior, and grade of the tumor according to the International Classification of Diseases for Oncology (ICD-O). This classification has evolved over the years with several editions and revisions that allow coding of newly defined cancer entities. Thus, as far as possible, it is recommended that registries adapt their coding criteria to the new editions and revisions of the ICD-O.

The recommended version of the ICD-O until the end of 2019 was the International Classification of Diseases for Oncology, 3rd edition, 1st revision (ICD-O-3.1) (15) and the second revision (ICD-O-3.2) which is not yet published is recommended to be used for tumors diagnosed on or after January 1, 2020 as reported on the IACR website (16).

The new versions of the ICD-O try to adapt as much as possible to the most recent versions of the WHO Classification of Tumours, which include morphological codes. In June 2022, a new version of the “WHO Classification of Tumours: Urinary and Male Genital Tumours” has been published (3). In some cases, the latest version of the ICD-O does not include a morphological category. For example, nested urothelial carcinoma is not covered by OCD-O-3.2. However, the latest edition of the WHO Classification of June 2022 indicates that it should be coded with code 8120/3.

*“All urothelial tumors must be coded according to the most recent version of the International Classification of Diseases for Oncology, 3rd Edition (ICD-O-3) (these classifications are almost equivalent to the WHO classification).”*

### Morphology, behavior and grade

#### a) Codes of the most frequent morphological categories when histology is available

Any of the morphological types can be found in any of the topographies of the urinary tract (renal pelvis, ureter, bladder, and urethra). Likewise, apart from the existing difficulties in determining the exact subtype of urothelial carcinoma, often the greatest difficulty is in determining the level of tumor invasion. This occurs because the evaluation of biopsies and transurethral resections of the bladder (TURB) can be extremely difficult for several reasons (9). First, proper pathology reporting is extremely dependent on the quality of the submitted material. Cautery artifact may hinder accurate staging at initial TURB for large tumors by understaging up to 6% of patients (17). Second, pathologists can have difficulty recognizing superficial invasion of the lamina propria and differentiating invasion of the muscularis propria from invasion of the muscularis mucosae (18).

When a tumor has been examined histologically but it has not been possible to determine the level of invasion, a dilemma arises in the cancer registry between coding behavior/2 (non-invasive carcinoma) or/3 (invasive carcinoma). In these situations, behavior/3 is never assigned by default, but only if there is a clinical impression of an invasive tumor (then use the code 8120/3). Otherwise (i.e. no obvious invasion on clinical/paraclinical examination), the code 8130/2 (non-invasive papillary tumor) must be used when the term papillary is mentioned in the pathology report or when the TURB report mentions papillary appearance. However, if the term papillary is not mentioned or there is no information about the appearance of the tumor, then it cannot be coded as papillary and code 8120/2 should be used. In this case the code pT is pTX (and not pTis), to avoid confusion with carcinoma *in situ*.

All *in situ* and invasive carcinomas should be coded as grade 3 even if the pathology report indicates “low grade” or does not indicate the grade. The reason is explained in section 2.e of these grading and coding criteria.

**Table:** 

*Tumor type*	*Morphology/Behavior*	*Grade*
*Non-invasive (papillary) urothelial carcinoma, low-grade*		*1*
*Non-invasive (papillary) urothelial carcinoma, high-grade*	8130/2*	*3*
*Non-invasive (papillary) urothelial carcinoma, grade unknown*		*9*
*Urothelial carcinoma (with histologic examination), but invasion cannot be assessed*		
*Papillary term mentioned or papillary appearance (exophytic lesion)*	8130/2**	
*Papillary term not mentioned or no information about appearance*	8120/2 ***	*1/3/9*
*The clinical impression is of invasive disease*	8120/3****	*3*
*Urothelial carcinoma in situ* (carcinoma *in situ*)	*8120/2*	3*****
*Invasive carcinoma, not otherwise specified (NOS) (1)*	*8010/3*	3*****
*Invasive urothelial carcinoma*	*8120/3*	3*****

*(1) Although most carcinomas of urinary tract are urothelial, there are also other carcinomas such as squamous or adenocarcinoma. Therefore, if urothelial or transitional cell is not specified on the pathological report, code “8010/3”. But if non-invasive urothelial carcinoma was previously diagnosed, record (code) as urothelial carcinoma (8120/3), provided that prostate carcinoma invading the urinary bladder is ruled out. Also, if the concept urothelial is in the tumor description, code 8120/3 even if not specified in the final diagnosis.*

(*) When the term “papillary” is not specified in the pathological report but the pathology report indicates an urothelial carcinoma with pTa stage, code 8130/2 (plus grade, if specified)

(**) In this case, code pTa.

(***) In this case the code pT is pTX (and not pTis), so as not to be confused with Carcinoma in situ.

(****) If the clinical impression is of invasive disease, then code with/3 behavior code and grade 3.

(*****) All in situ and invasive carcinomas must be recorded as high grade. Although the pathology report may indicate “low grade” or not indicate a grade, if it is an in situ or invasive tumor, it must be considered high grade.”

#### b) Codes when only cytological examination is available*

Although all cases should have histological examination, in a few cases only cytological examination can be found. This may be because the patient has not had a histologic examination or because the cancer registry does not have it available. In these cases, it is recommended to use the “Paris System reporting for urine cytology (19–22).

This System have the following seven diagnostic categories: 1. Non-diagnostic/Unsatisfactory; 2. Negative for high-grade urothelial carcinoma (NHGUC); 3. Atypical urothelial cells (AUC); 4. Suspicious for high-grade urothelial carcinoma (SHGUC); 5. High-grade urothelial carcinoma (HGUC); 6. Low-grade urothelial neoplasm (LGUN), and 7. Other: primary and secondary malignancies and miscellaneous lesion. Of these, only categories 4 and 5 should be considered as high-grade urothelial carcinomas.

In these high-grade tumors diagnosed by cytological examination only, a consensus has been agreed upon for high-grade urothelial carcinoma to be coded as behavior/2 although it was acknowledged there is a limited evidence base to support either this or coding to behavior code/3. In any case, in cancer registration if there is the clinical impression, e.g. with imaging, that the tumor is invasive then it should be coded with the behavioral code/3. In these cases, an effort should also be made to ascertain whether the tumor has a papillary appearance (8130) or not (8120) by reviewing the imaging.

Non-urothelial malignant cells may also be found on cytology. Evidently, in these cases, non-urothelial malignant cells seen on cytology should be coded according to the pathology report and clinical information. This would be the case, for example, for non-urothelial urinary tract tumors (squamous, glandular, Müllerian type, neuroendocrine, melanocytic, mesenchymal…) and metastases from tumors outside the urinary tract.

Of course, an effort should also be made to identify and code the exact topography of the tumor by radiology/imaging. If this is not known, topography 68.9 (Urinary tract, not otherwise specified (NOS) should be coded as the tumor can be located at any point between the renal pelvis and the urethra.

**Table:** 

*Cytology results*	Morphology*/Behavior **	*Grade*
*High grade urothelial carcinoma or “suspicious for high-grade urothelial carcinoma” (SHGUC of the Paris classification). (See ANNEX 2, section “Paris System reporting for urine cytology”, paragraph “Behavior of high grade tumors diagnosed by cytology only”).*	*8130/2 (papillary appearance) or 8120/2*	*3*

(*) If you only have cytological examination, try to find out if the tumor has a papillary appearance (8130) or not (8120) by reviewing the imaging.

(**) If the clinical impression (e.g. scans) is of invasive disease, then code with/3 behavior code.

*Non-urothelial malignant cells seen on cytology should be coded according to the pathology report and clinical information.*

*If the topography of the tumor is highlighted on radiology/imaging, code the specific site. Otherwise, code the topography C68.9 (urinary tract, NOS).”*

#### c) Codes when only non-microscopic confirmation is available (histo/cytopathological evidence unavailable)

In other rare situations, neither histological nor cytopathological evidence is available. In these cases, only tumors with a clinically malignant appearance can be recorded, which can be coded as 8000/3 because the morphologic result is not available, and grade 9 because it is also unknown. If the tumor has no malignant appearance or its appearance of malignancy is doubtful, it is not necessary to register it and, if it is decided to register it, code it as 8000/1 grade 9.

“When histo/cytopathological evidence is unavailable but clinical appearance is confirmed by the clinician, use the following codes.

**Table:** 

*Tumor type*	*Morphology/Behavior*	*Grade*
*No microscopic confirmation: Tumor clinically malignant*	*8000/3*	*9*
*No microscopic confirmation: Tumor NOS*	Do not record*	

(*) If recorded, code: 8000/1 Grade 9.”

#### d) Codes of behavior for unknown level of invasion

When there is a histologic examination but the exact level of invasion is unknown, it is usually because either subepithelial connective tissue or muscularis propria is not present in the specimens received by the pathologist. In either of these cases the first thing to do, if possible, is to consult the pathologist for advice/assessment.

In case of urothelial papilloma, papillary urothelial neoplasms of low malignant potential (PUNLMP) or urothelial proliferation of uncertain malignant potential, the recommendation is not to register these entities as already mentioned in the point “1.1. Types of tumors to be included” of the Criteria for the inclusion (registration) of urothelial tumors.

What should be done when subepithelial connective tissue is not present? As the lack of subepithelial connective tissue does not preclude the diagnosis of non-invasive carcinomas, if it diagnosed as a “Non-invasive papillary urothelial carcinoma” or a “Carcinoma *in situ*” code behavior/2. However, if morphological characteristics are not specified, code behavior/2 because it is the maximum aggressiveness that can be assumed (behavior/3 should never be assigned by default). In relation to the morphology, the code to use depends on the appearance at endoscopy: 8120 (no papillary appearance) or 8130 (papillary appearance).

And what to do when muscularis propria is not present? If sub-epithelial connective tissue is invaded, code behavior/3. But, otherwise, code behavior/2 according to the morphological characteristics (papillary or not).

**“d1) “Subepithelial connective tissue” is not present in resection.**

First of all, ask for pathologist assessment. If it is not possible or the pathologist can’t give an answer:

− If “Urothelial papilloma”:/0 (there is no recommendation to record this tumor).
− If “Papillary urothelial neoplasm of low malignant potential (PUNLMP)”:/1 (there is no recommendation to record this tumor but if it is recorded, code 8130/1 without grade and pT) (some pathologists can erroneously code pTa in PUNLMP. pTa should be used only in carcinomas).
− If “Urothelial proliferation of uncertain malignant potential”:/1 (there is no recommendation to record this entity).
*− If “Non-invasive papillary urothelial carcinoma” or “Carcinoma *in situ*”:/2*
*− If morphological characteristics are not specified:/2 (Codify morphology 8120 (no papillary appearance) or 8130 (papillary appearance) depending on the appearance at endoscopy).*

d2) *“Muscularis propria” is not present in resection.*

*First of all, ask for pathologist assessment. If it is not possible or the pathologist can’t give an answer:*

*− If sub-epithelial connective tissue is invaded:/3.*
*− Otherwise, code behavior/2 (according to the morphological characteristics).”*

#### e) Grade

Grade registration is especially important for the non-invasive papillary urothelial carcinomas where it is necessary to distinguish between the high-grade (code 3) and the low-grade (code 1) tumors. As in 2004, the 2016 WHO Classification recommends the use of the grading classification first put forth by ISUP in 1997 (2). This 2-tiered grading system—high versus low grade—is intended to simplify clinical decision making in daily practice over the 3-tiered 1973 system. It also provides congruence between histology and cytology reports, and highlights the prompt therapeutic requirement for all high-grade lesions (flat or papillary) (23). Moreover this system does not outperform the 1973 system in prognostic value, but shows higher reproducibility (24). If the pathology report does provide tumor grades according to both 2016 and 1973 systems or does not indicate whether the tumor is low grade or high grade, but rather indicates the grade based on the three categories of level 1, 2 and 3, the following table of these Recommendations shows the correspondence between the two classifications. As a result, code 2 will no longer be used to code the grade.

In relation to the invasive urothelial carcinomas, the overwhelming majority of invasive urothelial carcinomas are high grade (25). However, some variants (e.g. large nested variant of urothelial carcinoma) may present a “pseudo-benign” (deceptively bland) appearance, but this appearance is misleading, since these variants have a poor outcome (26–28). On this basis, all invasive urothelial tumors should be recorded as ‘Grade 3’.

“*Codes according to the description in the pathological report:*

**Table:** 

*Description in the pathology report*	*Code*
*Grade 1*	*Low grade (1)*
*Grade 1/2 (low grade or no grade mentioned)*	*Low grade (1)*
*Grade 2 low grade*	*Low grade (1)*
*Grade 2 high grade*	*High grade (3)*
*Grade 2/3 (high grade or no grade mentioned)*	*High grade (3)*
*Grade 3*	*High grade (3)*

### Codes for urothelial carcinomas with other morphological terms

Urothelial carcinoma has long been known to have a remarkable propensity for divergent differentiation (29), which is seen most commonly in association with high-grade and locally advanced disease. Common morphologic manifestations of divergent differentiation are along squamous and secondly glandular lines, but also along trophoblastic lines. Around 10% of cases have multiple mixed histologic types (30).

This remarkable propensity for morphological diversity is due both to divergent differentiation and to the existence of histological subtypes. Much literature has been devoted to the characterization and definition of histological entities, but only few prospective data exist (31). Recently, molecular classification (i.e. on basis of expression and genetic alterations) has enriched our understanding of bladder cancer and provided us with a new framework for stratification and assessing response to different therapy regimens (32). It is important to understand that when talking about divergent differentiation or subtypes, a therapeutic implication exists. Therefore, the pathologist must be aware of the diagnostic criteria and accurately report them (8).

**Urothelial carcinoma with squamous cell divergent differentiation (with an squamous component):** We must differentiate pure squamous cell carcinoma from urothelial carcinoma with squamous cell divergent differentiation (with an epidermoid component) because they are a different tumor type and are treated differently (7). Therefore, we must code the morphology of the first one as 8070 and the latter as 8120.*”*

***a) Urothelial cell carcinoma with epidermoid component (squamous divergent differentiation): 8120***

*Code squamous carcinoma only if it is a pure squamous carcinoma: 8070 “Pure squamous carcinomas” should be registered separately from urothelial carcinomas because they are a different tumor type from urothelial carcinomas and are treated differently (1, 2), even if the 2004 International Rules for Multiple Primary Cancers include this two tumors in the same morphology group.*

**Urothelial carcinoma with an adenocarcinomatous component (glandular divergent differentiation):** The same applies to the urothelial carcinoma with an adenocarcinomatous component (glandular divergent differentiation). So, we must code as adenocarcinoma only if it is a pure adenocarcinoma (8140) and for urothelial cell carcinoma with adenocarcinomatous component, the code must be 8120.

**b) Urothelial cell carcinoma with adenocarcinomatous component (glandular divergent differentiation): 8120**

*Code adenocarcinoma only if it is a pure adenocarcinoma: 8140*

*“Pure adenocarcinomas” should be registered separately from urothelial carcinomas because they are a different tumor type from urothelial carcinomas.*

**Urothelial cell carcinoma subtypes and ICD-O-3 specific code:** ICD-O-3 and the 2016 WHO Classification, contains some subtype codes for urothelial tumors. These codes should be used whenever they are reported in pathology reports. These codes are: micropapillary: 8131, lymphoepithelioma-like: 8082, sarcomatoid: 8122, giant cell: 8031 and undifferentiated: 8020.

***c) Urothelial cell carcinoma subtypes and ICD-O-3 specific code*** *(new specific codes may appear in subsequent versions of ICD-O/WHO Classification):*

*− Micropapillary: 8131*
*− Lymphoepithelioma-like: 8082*
*− Sarcomatoid: 8122*
*− Giant cell: 8031*
*− Undifferentiated: 8020*

**Urothelial cell carcinoma without specific subtype in ICD-O-3 classification:** The other subtypes of urothelial carcinomas without a specific morphological code in the ICD-O-3 classification (e.g. nested, microcystic, plasmacytoid, signet ring cell, diffuse, lipid-rich, clear-cell) must be coded as 8120. However, it is possible that some of these subtypes may have specific codes in subsequent versions of ICD-O/WHO Classification. If this occurs, it is recommended to use the new codes that appear. This is in fact already the case since the fifth edition of the WHO classification was recently published (2022), shortly after the release of these recommendations. This new classification assigns the morphological code 8122/3 not only to sarcomatoid urothelial tumors but also to plasmocytoid, signet ring cell and diffuse urothelial tumors (3).

***d) Urothelial cell carcinoma without specific subtype in ICD-O-3 classification (e.g. nested, microcystic, plasmacytoid, signet ring cell, diffuse, lipid-rich, clear-cell)*** *(some of these may have specific codes in subsequent versions of ICD-O/WHO Classification): 8120*

**Urothelial cell carcinoma with neuroendocrine component (neuroendocrine differentiation):** A very different case is that of neuroendocrine tumors. Neuroendocrine tumors are classified into well-differentiated neuroendocrine tumor and neuroendocrine carcinoma which includes both large- and small-cell neuroendocrine carcinoma. Whereas well-differentiated neuroendocrine tumors occur in pure form, neuroendocrine carcinomas are often admixed with some form of non-neuroendocrine carcinoma that is most frequently urothelial carcinoma (33). Both large- and small-cell neuroendocrine carcinomas can arise within the bladder. Large-cell neuroendocrine carcinoma is extremely uncommon whereas the incidence of small-cell neuroendocrine carcinoma is only 0.5-1.0% (34). The cell of origin of neuroendocrine carcinoma is unclear (35). Microscopically, large-cell neuroendocrine carcinomas are usually high-grade and poorly differentiated. Approximately 50% of cases of small-cell neuroendocrine carcinomas show an admixture of small-cell neuroendocrine carcinoma with non-small-cell carcinoma components (33, 36) and the ratio of neuroendocrine and non-neuroendocrine components may vary and the amount of the neuroendocrine carcinoma component may be important to outcomes.

The term “Neuroendocrine carcinoma” should be used in all tumors with small or large cell neuroendocrine histology in any proportion of the tumor (37). Recording the histological tumor type using the 2016 WHO classification is a required element as this parameter often has prognostic and therapeutic significance. Therefore, the code assigned to the tumor morphology should most accurately reflect the pathological diagnosis from among the following: 8041 (small cell neuroendocrine carcinoma), 8013 (large cell neuroendocrine carcinoma), 8045 (small and large cell carcinoma), 8240 (neuroendocrine carcinoma well-differentiated or low-grade), 8249 (neuroendocrine carcinoma moderately-differentiated or high grade) and 8246 (neuroendocrine carcinoma, NOS).

A tumor is classified as urothelial carcinoma if there is any urothelial differentiation [including associated urothelial carcinoma *in situ* (CIS)], with any other types present reported with an estimated percentage. Thus, a carcinoma showing 20% urothelial differentiation and 80% glandular differentiation should be reported under the histological tumor type “Urothelial carcinoma”. An exception to this rule is for cases with any amount of neuroendocrine component (small cell neuroendocrine carcinoma or large cell neuroendocrine carcinoma) where classification is now in the neuroendocrine tumor category. Thus, a mixed tumor with 30% small cell neuroendocrine carcinoma and 70% urothelial carcinoma should be reported under the histological tumor type as neuroendocrine tumor (small cell neuroendocrine carcinoma). This is a controversial issue, as reflected by the different approaches recommended by WHO 2016 in chapters on the neuroendocrine tumors and urothelial carcinoma variants. The International Collaboration on Cancer Reporting (ICCR) recommends the latter approach but recognizes that the percentage of the neuroendocrine component could inform patient management, particularly with newer treatment modalities such as immunotherapy.

**e) Urothelial cell carcinoma with neuroendocrine component (neuroendocrine differentiation):**

Always encode neuroendocrine carcinoma independently of the amount of the neuroendocrine component (See Annex 2: Comments. Neuroendocrine tumors).

− Small cell neuroendocrine carcinoma: 8041
*− Large cell neuroendocrine carcinoma: 8013*
*− Composite small and large cell neuroendocrine carcinoma: 8045*
*− Neuroendocrine carcinoma well-differentiated or low-grade NET: 8240*
*− Neuroendocrine carcinoma moderately-differentiated or high-grade NET: 8249*
*− Neuroendocrine carcinoma, NOS: 8246”*

### Non-urothelial specific carcinomas

Unlike urothelial tumors with squamous, glandular or other types of differentiation, there are non-urothelial tumors of the urinary tract such as (pure) adenocarcinomas, (pure) squamous carcinomas, and neuroendocrine, melanocytic, mesenchymal or lymphoid tumors (38) which must be recorded separately from urothelial tumors following the general criteria for other tumors.

**Table 1** summarizes the main criteria of inclusion (according to invasion, grade and existence of progression). For each site (right and left pelvis, right and left ureter, bladder and urethra), this table summarizes, which tumors should be registered by application of rules 2 to 8. In summary: after recording the first tumor (/2 or/3) of each site, only record subsequent tumors that represent progression, according to the grouping of categories (columns 1, 2, 3, 4 and 5).

**Table 1:** Summary table of main criteria of inclusion (according to invasion, grade and existence of progression).

STEPS of PROGRESSION				
1. Non-invasive low grade/grade unknown	2. Non-invasive high-grade or invasion cannot be assessed	3. *In situ*	4. Invasive (T1)	5. Invasive (T2+)
**8130/2 G1** **or** **8130/2 G9**	**8130/2 G3** **or** **8120 or 8130/2 G3** **or** **8120/2 G3**	**8120/2 G3**	**8010/3 G3** **or** **8120/2 G3** **or** **8000/3 G9**	**8010/3 G3** **or** **8120/3 G3** **or** **8000/3 G9**
Non-invasive Papillary Carcinoma, Low Grade or Non-invasive Papillary Carcinoma, Grade unknown	Non-invasive Papillary Carcinoma, High Grade or High grade urothelial carcinoma on cytology or Suspicious for high grade urothelial carcinoma on cytology	Urothelial Carcinoma *In situ* or Urothelial carcinoma with histologic examination but invasion cannot be assessed	Invasive carcinoma NOS or Invasive urothelial carcinoma or No microscopic confirmation: Tumour clinically malignant	Invasive carcinoma NOS or Invasive urothelial carcinoma or No microscopic confirmation: Tumour clinically malignant

### Coding the Basis of Diagnosis

Considering the current methods for diagnosing urothelial tumors, the possible usable codes are as follows. Evidently, as with all other cancers, if the cancer registry only has a record of the case by death certificate, the code to be used is “0” (Death certificate only).

- Histology (Biopsy or surgical resection or autopsy specimen) …………………………………………… 7
- Cytology only (urine) ………………………………… 5
- Only imaging or cystoscopy without biopsy or autopsy without a tissue diagnosis …………………………․ 2
- Death certificate only ………………………………… 0

In case of doubt, see the ENCR Recommendations on Basis of Diagnosis. It should be noted that the current recommendations on the basis of diagnosis were distributed in 1999, and it is likely that new ENCR recommendations on this subject will be published soon. Finally, if in the future the diagnostic methods for urothelial tumors are expanded and, consequently, these codes are modified or expanded, it is recommended to follow the modifications that may be defined by the ENCR.

### Coding stage

Stage at diagnosis is one of the most important prognostic factors for the vast majority of tumors and this is also true for urothelial tumors (39). For this reason, survival analyses should be performed not only by sex and age but also by stage in order to distinguish whether differences in survival over time or between populations are due to a different distribution of cases by stage or to differences in cancer care.

In urothelial carcinomas, it is important to distinguish tumor invasion of the smaller, discontinuous, slender smooth muscle fibers of the muscularis mucosae (T1) from invasion of the larger, compact bundles of the muscularis propria (40, 41) and, as already commented, pathologists can have difficulty recognizing focal, superficial invasion of the lamina propria and differentiating invasion of the muscularis propria from invasion of the muscularis mucosae -ie, stage T1 from T2, which has immense implications for patient care (18).

On the other hand, although the combination of morphological, behavioral and grade codes are sufficient to distinguish between carcinomas *in situ* (CIS) and noninvasive papillary carcinomas, recording the T category of noninvasive tumors (pTis or pTa) in cancer registries validates the correctness of the data.

***“Record “TNM-stage” (1, 4) whenever possible and, at least the “T-category”***.

*This is important to allow Tis tumors to be easily distinguished from other tumors with behavior/2.”*

## Recommendations for reporting urothelial tumors

Due to the complexity of urothelial tumors, these Recommendations are mostly devoted to recording criteria (registration, coding and classification). However, recommendations for the reporting of these tumors are also important for data comparability and are discussed below.

In order to follow the IARC/IACR/ENCR “International Rules for Multiple Primary Cancers” for computing incidence, only the first urothelial tumor regardless of the behavioral code (/2 or/3) should be counted. This will ensure incidence comparability between registries

The most important fact to note is that following the recommendations for recording provides the raw data that can be analyzed later. By doing so, data from cancer registry databases can be used to perform multiple analyses as part of local cancer surveillance and service assessment or can be transmitted for National, European or International projects.

The objectives of international projects can be very varied, so the “data call protocol” from international projects should define very accurately the criteria for inclusion of the data to be submitted and should also explain in detail how the data will be analyzed for incidence and survival estimations.

The following two examples show how the objectives and, consequently, the use of data for analysis can vary:

1. Counting the incidence of urinary bladder cancer: will a patient’s first urothelial tumor be counted regardless of whether it is invasive or non-invasive, or will only invasive urothelial tumors be counted? Will non-urothelial bladder tumors also be included in the calculation?

2. Urinary bladder cancer survival computation: will the first tumor from any patient regardless of her behavior be included in the analysis or will only invasive tumors be considered?

**Table 2** summarizes the general principles and criteria for inclusion (registration), and list of coding issues in the 2022 ENCR recommendations on urothelial tumours.

**Table 2:** Summary of the general principles and criteria of inclusion (registration), and list of coding issues in the 2022 ENCR recommendations on urothelial tumours.

General principles
- These recommendations should be applied to the pure urothelial carcinomas, to urothelial carcinomas with divergent differentiation, and to all other variants.
- Do everything possible to have access to pathological examinations (reports)
- Synchronous urothelial tumors are considered to be all those in the same site that appear in a period of less than or equal to 4 months.
**Recommendations for recording urothelial tumors**
**Criteria for inclusion**
- Types of tumors to be included: all invasive and non-invasive urothelial carcinomas including those without histological confirmation.
- Record all synchronous or metachronous urothelial tumors in different sites and laterality.
- Record progressions of the same three-digit sites.
- Do not record recurrences.
- In case of synchronous tumors of the same three-digit site and laterality, record only the most aggressive one
- In case of some synchronous bladder tumors of different subsite with the same level of progression, code as C67.8
- In case of some synchronous urothelial tumors of different three-digit site, code as C67.8.
- Record all the tumors of each side of each three digit site following the previous rules.
- If a patient has been previously diagnosed with an urothelial tumor(s) when resident outside the registration area, record all of them (the ones occurring outside the area of registration and the ones diagnosed being resident in the area of the registry) according to previous rules.
- If a patient has been diagnosed with one or more urothelial tumor(s) before the operation period of the registry, record all their tumors (the ones diagnosed before and the one diagnosed after first date of operation of the registry) according to previous rules.
**Coding**
- Code according to the most recent version of the International Classification of Diseases for Oncology
- Codes of the most frequent morphological categories when histology is available
- Codes when only cytological examination is available
- Codes when only non-microscopic confirmation is available
- Codes of behavior for unknown level of invasion
- Coding of Grade
- Codes for urothelial carcinomas with other morphological terms
Urothelial carcinoma with squamous cell divergent differentiation
Urothelial carcinoma with an adenocarcinomatous component
Urothelial cell carcinoma subtypes and ICD-O-3 specific code
Urothelial cell carcinoma without specific subtype in ICD-O-3 classification
- Non-urothelial specific carcinomas
- Coding the Basis of Diagnosis
- Coding stage
**Recommendations for reporting urothelial tumors**
- The Recommendations for recording provide the raw data which can be subsequently analysed.
- Follow IARC/IACR rules to calculate incidence (according to the “International Rules for Multiple Primary Cancers”)
- At the local level, analyze the data recorded and coded with the new Recommendations according to the defined objectives.
- In international projects, define very precisely the inclusion criteria for the data to be submitted and explain in detail how the data will be analyzed for incidence and survival estimates.

In conclusion, due to the great variability in the criteria for registration, coding and reporting of urothelial tumors among the different cancer registries, it is very difficult to determine the quantitative impact on incidence and survival rates of these new “ENCR Recommendations on the registration and reporting of urothelial tumors of the urinary tract”. This would only be possible with the performance of a prospective study performed by all cancer registries applying both the old and the new criteria and evaluating the differences. These ENCR Recommendations imply a higher workload for the registry teams but will provide the framework to ensure comparability of outcomes for this tumor type across cancer registries in Europe and to enable a broader spectrum of analysis of incidence, survival and prevalence data for urothelial tumors. In the medium-term, an evaluation to review if the updated recommendations had any impact on the incidence and the quality of registered of urothelial tumors by registries would be desirable.

Finally, the rules we propose for the registration of urothelial tumors, which constitute the most complex example of multiple, recurrent or progressive tumors, could be extended to tumors of other locations that may present these characteristics, in particular tumors for which screening programs exist, such as breast or colon-rectal tumors.

## Data availability statement

The original contributions presented in the study are included in the article/supplementary material. Further inquiries can be directed to the corresponding authors.

## Author contributions

JG, LM, DP, ME, RT, AW, CM and LN contributed to the development of the ENCR Recommendations. OV critically reviewed the ENCR Recommendations. The first draft of the manuscript was written by JG, LM and DP. All authors contributed to the article and approved the submitted version.

## Conflict of interest

The authors declare that the research was conducted in the absence of any commercial or financial relationships that could be construed as a potential conflict of interest.

## Publisher’s note

All claims expressed in this article are solely those of the authors and do not necessarily represent those of their affiliated organizations, or those of the publisher, the editors and the reviewers. Any product that may be evaluated in this article, or claim that may be made by its manufacturer, is not guaranteed or endorsed by the publisher.

## References

[1] Pheby D Martínez C Roumagnac M Schouten L ENCR Working Group . ENCR recommendations for coding bladder cancers (1995). ENCR. Available at: https://www.encr.eu/sites/default/files/pdf/bladeng.pdf (Accessed July 15, 2022).

[2] Moch H Humphrey PA Ulbright TM Reuter VE . WHO classification of tumors of the urinary system and Male genital organs. 4th ed. world health organization classification of tumors. Lyon, France: IARC Press (2016).

[3] Tsuzuki T Compérat EM Netto GJ Raspollini PM Moch H Srigley JR . Tumours of the urinary tract, in: WHO classification of tumours editorial board. urinary and male genital tumours (2022). Lyon (France: International Agency for Research on Cancer. Available at: https://tumourclassification.iarc.who.int/ (Accessed July 15, 2022).

[4] Ferlay J Kraywinkel K Rous B Znaor A . Chapter 3: Classification and coding, in: Cancer incidence in five continents, vol. XI (electronic version) (2017). Lyon: International Agency for Research on Cancer. Available at: https://ci5.iarc.fr (Accessed July 15, 2022).

[5] Daubisse-Marliac L Grosclaude P Carulla M Parada D Vilardell L Ameijide A . Registration of urothelial tumors in cancer registries: How to improve and make it more useful? Int J Environ Res Public Health (2022) 19:2714. doi: 10.3390/ijerph19052714 35270406 PMC8910388

[6] Eden M Daubisse-Marliac L Galceran J Martos C Neamtiu L Parada D . ENCR recommendations. recording and reporting of urothelial tumors of the urinary tract. European network of cancer registries (2022). Available at: https://www.encr.eu/sites/default/files/Recommendations/ENCR%20Recommendation_UT_Jun2022_EN.pdf (Accessed July 15, 2022). 10.3389/fonc.2022.1046239 PMC9727225 36505871

[7] Audenet F Attalla K Sfakianos JP . The evolution of bladder cancer genomics: What have we learned and how can we use it? Urol Oncol (2018) 36:313–20. doi: 10.1016/j.urolonc.2018.02.017 29573965

[8] Park S Reuter VE Hansel DE . Non-urothelial carcinomas of the bladder. Histopathology (2019) 74:97–111. doi: 10.1111/his.13719 30565306

[9] Compérat E Oszwald A Wasinger G Hansel DE Montironi R van der Kwast T . Updated pathology reporting standards for bladder cancer: biopsies, transurethral resections and radical cystectomies. World J Urol (2022) 40:915–27. doi: 10.1007/s00345-021-03831-1 PMC8994708 34554298

[10] International Agency for Research on Cancer World Health Organization International Association of Cancer Registries European Network of Cancer Registries . International rules for multiple primary cancers (ICD-O third edition). internal report no. 2004/02 (2004). Lyon: International Agency for Research on Cancer. Available at: http://www.iacr.com.fr/images/doc/MPrules_july2004.pdf (Accessed July 15, 2022).

[11] Cambier S Sylvester RJ Collette L Gontero P Brausi MA van Andel G . EORTC nomograms and risk groups for predicting recurrence, progression, and disease-specific and overall survival in non-muscle-invasive stage Ta-T1 urothelial bladder cancer patients treated with 1-3 years of maintenance bacillus calmette-guérin. Eur Urol (2016) 69:60–9. 10.1016/j.eururo.2015.06.045 10.1016/j.eururo.2015.06.045 26210894

[12] van Rhijn BWG Burger M Lotan Y Solsona E Stief CG Sylvester RJ . Recurrence and progression of disease in non-muscle-invasive bladder cancer: from epidemiology to treatment strategy. Eur Urol (2009) 56:430–42. doi: 10.1016/j.eururo.2009.06.028 19576682

[13] Guo C Czerniak B . Bladder cancer in the genomic era. Arch Pathol Lab Med (2019) 143:695–704. doi: 10.5858/arpa.2018-0329-RA 30672335

[14] Farrow GM Utz DC Rife CC . Morphological and clinical observations of patients with early bladder cancer treated with total cystectomy. Cancer Res (1976) 36:2495–501. 1277156

[15] World Health Organization . International classification of diseases for oncology, third edition, first revision. Geneva: World Health Organization (2013).

[16] ICD-O-3.2 (2021). Available at: http://www.iacr.com.fr/index.php?option=com_content&view=category&layout=blog&id=100&Itemid=577 (Accessed July 15, 2022).

[17] Truong M Liang L Kukrej J O’Brien J Jean-Gilles J Messing E . Cautery artifact understages urothelial cancer at initial transurethral resection of large bladder tumours. Can Urol Assoc J (2017) 11:E203–6. doi: 10.5489/cuaj.4172 PMC5426942 28503235

[18] Paner GP Brown JG Lapetino S Nese N Gupta R Shen SS . Diagnostic use of antibody to smoothelin in the recognition of muscularis propria in transurethral resection of urinary bladder tumor (TURBT) specimens. Am J Surg Pathol (2010) 34:792–9. doi: 10.1097/PAS.0b013e3181da7650 20421781

[19] Barkan GA Wojcik EM Nayar R Savic-Prince S Quek ML Kurtycz DF . The Paris system for reporting urinary cytology: The quest to develop a standardized terminology. Adv Anat Pathol (2016) 23:193–201. doi: 10.1097/PAP.0000000000000118 27233050

[20] VandenBussche CJ . A review of the Paris system for reporting urinary cytology. Cytopathology (2016) 27:153–6. doi: 10.1111/cyt.12345 27221750

[21] Rosenthal DL Wojcik EM Kurtycz DFI eds. The Paris system for reporting urinary cytology. Switzerland: Springer International Publishing (2016).

[22] Owens CL VandenBussche CJ Burroughs FH Rosenthal DL . A review of reporting systems and terminology for urine cytology. Cancer Cytopathol (2013) 121:9–14. doi: 10.1002/cncy.21253 23192885

[23] Compérat E Amin M Reuter V . Reply re: Murali varma, Brett delahunt, theodorus van der kwast. grading noninvasive bladder cancer: World health organisation 1973 or 2004 may be the wrong question. two decades of world health organisation/international society of urological pathology bladder cancer grading: time to reflect on accomplishments and plan refinement in the molecular era, not regress to readoption of a 45-year-old classification. Eur Urol (2019) 76:416–7. doi: 10.1016/j.eururo.2019.07.017 31350070

[24] Soukup V Čapoun O Cohen D Hernández V Babjuk M Burger M . Prognostic performance and reproducibility of the 1973 and 2004/2016 world health organization grading classification systems in non-muscle-invasive bladder cancer: a European association of urology non-muscle invasive bladder cancer guidelines panel systematic review. Eur Urol (2017) 72:801–13. doi: 10.1016/j.eururo.2017.04.015 28457661

[25] Nishiyama N Kitamura H Maeda T Takahashi S Masumori N Hasegawa T . Clinicopathological analysis of patients with non-muscle-invasive bladder cancer: prognostic value and clinical reliability of the 2004 WHO classification system. Jpn J Clin Oncol (2013) 43:1124–31. doi: 10.1093/jjco/hyt120 23980236

[26] Amin MB Smith SC Reuter VE Epstein JI Grignon DJ Hansel DE . Update for the practicing pathologist: The international consultation on urologic disease-European association of urology consultation on bladder cancer. Mod Pathol (2015) 28:612–30. doi: 10.1038/modpathol.2014.158 PMC5009623 25412849

[27] Lopez-Beltran A Henriques V Montironi R Cimadamore A Raspollini MR Cheng L . Variants and new entities of bladder cancer. Histopathology (2019) 74:77–96. doi: 10.1111/his.13752 30565299

[28] McKenney JK . Precursor lesions of the urinary bladder. Histopathology (2019) 74:68–76. doi: 10.1111/his.13762 30565304

[29] Grace DA Winterm CC . Mixed differentiation of primary carcinoma of the urinary bladder. Cancer (1968) 21:1239–43. doi: 10.1002/1097-0142(196806)21:6<1239::AID-CNCR2820210627>3.0.CO;2-E 4296749

[30] Wasco MJ Daignault S Zhang Y Kunju LP Kinnaman M Braun T . Urothelial carcinoma with divergent histologic differentiation (mixed histologic features) predicts the presence of locally advanced bladder cancer when detected at transurethral resection. Urology (2007) 70:69–74. doi: 10.1016/j.urology.2007.03.033 17656211

[31] Veskimäe E Espinos EL Bruins HM Yuan Y Sylvester R Kamat AM . What is the prognostic and clinical importance of urothelial and nonurothelial histological variants of bladder cancer in predicting oncological outcomes in patients with muscle-invasive and metastatic bladder cancer? a European association of urology muscle invasive and metastatic bladder cancer guidelines panel systematic review. Eur Urol Oncol (2019) 2:625–42. doi: 10.1016/j.euo.2019.09.003 31601522

[32] Kamoun A de Reyniès A Allory Y Sjödahl G Robertson AG Seiler R . A consensus molecular classification of muscle-invasive bladder cancer. Eur Urol (2020) 77:420–33. doi: 10.1016/j.eururo.2019.09.006 PMC7690647 31563503

[33] Abrahams NA Moran C Reyes AO Siefker-Radtke A Ayala AG . Small cell carcinoma of the bladder: A contemporary clinicopathological study of 51 cases. Histopathology (2005) 46:57–63. doi: 10.1111/j.1365-2559.2004.01980.x 15656887

[34] Trias I Algaba F Condom E Español I Seguí J Orsola I . Small cell carcinoma of the urinary bladder. presentation of 23 cases and review of 134 published cases. Eur Urol (2001) 39:85–90. doi: 10.1159/000052417 11173944

[35] Wang X MacLennan GT Lopez-Beltran A Cheng L . Small cell carcinoma of the urinary bladder-histogenesis, genetics, diagnosis, biomarkers, treatment and prognosis. Appl Immunohistochem Mol Morphol (2007) 15:8–18. doi: 10.1097/01.pai.0000213106.12731.d7 17536302

[36] Grignon DJ Ro JY Ayala AG Shum DT Ordoñez NG Logothetis CJ . Small cell carcinoma of the urinary bladder. a clinicopathologic analysis of 22 cases. Cancer (1992) 69:527–36. doi: 10.1002/1097-0142(19920115)69:2<527::AID-CNCR2820690241>3.0.CO;2-7 1309435

[37] Varma M Srigley JR Brimo F Compérat E Delahunt B Koch M . Dataset for the reporting of urinary tract carcinoma-biopsy and transurethral resection specimen: recommendations from the international collaboration on cancer reporting (ICCR). Mod Pathol (2019) 33:700–12. doi: 10.1038/s41379-019-0403-9 31685965

[38] Humphrey PA Moch H Cubilla AL Ulbrigth TM Reuter VE . The 2016 WHO classification of tumors of the urinary system and Male genital organs–part b: Prostate and bladder tumors. Eur Urol (2016) 70:106–19. doi: 10.1016/j.eururo.2016.02.028 26996659

[39] Magers MJ Lopez-Beltran A Montironi R Williamson SR Kaimakliotis HZ Cheng . Staging of bladder cancer. Histopathology (2019) 74:112–34. doi: 10.1111/his.13734 30565300

[40] Paner GP Ro JY Wojcik EM Venkataraman G Datta MW Amin MB . Further characterization of the muscle layers and lamina propria of the urinary bladder by systematic histologic mapping: implications for pathologic staging of invasive urothelial carcinoma. Am J Surg Pathol (2007) 31:1420–9. doi: 10.1097/PAS.0b013e3180588283 17721199

[41] Ro JY Ayala AG el-Naggar A . Muscularis mucosa of urinary bladder. importance for staging and treatment. Am J Surg Pathol (1987) 11:668–73. doi: 10.1097/00000478-198709000-00002 3631381

